# The South London Hospital for Women

**Published:** 1913-06-14

**Authors:** 


### The Hospital

The Workers' Newspaper of Administrative Medicine and Institutional Life, Administration, National Insurance and Health.

No. 1407, Vol. LIV. SATURDAY, JUNE 14, 1913. PRICE ONE PENNY.

### THE SOUTH LONDON HOSPITAL FOR WOMEN.

Some months ago a project was publicly put forward, as we noted at the time, for the creation in South London of a General Women's Hospital, staffed entirely by medical women, and consisting of both general and private wards and an out-patient department. The scheme has been actively promoted since then, and is already sufficiently advanced to leave no doubt of its eventual accomplishment. By the courtesy of the Secretary we have been furnished with an account of what has been done and of what remains still to do; and, although the latter item is considerable, the start which has been made is highly creditable considering the short time since the inception of the idea.

The out-patient department was opened on April 2 last by Viscountess Castlereagh. For this purpose a house was taken in Newington Causeway, and adapted for the purpose. The attendance of patients is rapidly increasing, but it is hardly to be expected that in so short a time the clinic can have got that reputation amongst the hospital public which some of the older institutions enjoy. Subscribers' letters have not been issued, and no charge is made for treatment; but patients are required to contribute threepence a week towards the cost of their medicines. An almoner is provided for in the scheme, but the report does not state whether she has yet been appointed. Presumably the department is staffed by the two assistant physicians and two assistant surgeons who have been appointed.

The arrangements for the in-patient department are naturally not so well forward. To begin with, the contemplated hospital will cost £55,000 according to the estimate, a sum which cannot be raised in a moment. Fortunately for the success of this part of the scheme, some anonymous donors have provided the very large sum of £35,000, free of all conditions. The appeals which have been issued have resulted in the provision of an additional £5,000, so that £15,000 is still required to open the buildings completely equipped and free from debt. The committee have decided to proceed with the plans, trusting to the future for the collection of this sum. It is hoped to lay the foundation-stone this month, and to complete the building next summer. The site chosen is one of three acres at Clapham Common, and will allow of future expansion if and when necessary. The plans provide for general wards for those of the ordinary hospital class, who, if they can afford it, will be expected to contribute small sums towards the expenses of their treatment; and of private wards for patients at an inclusive cost of from one to three guineas a week. These wards are intended for those of small means who cannot afford a private nursing home, or to pay a nurse in their own homes, and yet are ineligible for the ordinary hospital wards.

The committee in their appeal for help in raising the £15,000 required explain that unless this sum can be raised they will be compelled to forego either one or two wards, or the nurses' home, or all three. We sincerely hope that if any such curtailment of the immediate programme does become necessary, a great effort will be made not to omit the nurses' home: for our own part we should contemplate the deferment of one or two wards with much greater equanimity than any makeshift arrangements for the accommodation of the nurses. And there is another argument, the financial one, which points in the same direction. As yet the subscription list, as opposed to donations to the building fund, of the new venture is of very modest dimensions indeed; and for some little time legacies are not likely to be of frequent occurrence. The current expenses of the in-patient department will be very considerable, and the committee will have all their work cut out to make ends meet. Therefore, a temporary curtailment of the ward-building scheme will save much annual expense during the early stages of the institution, when some financial stringency is to be expected. On the other hand, if nurses' quarters are not erected, no administration expense is thereby saved, because the nurses have to be boarded somehow; and any plan for accommodating them outside the hospital is likely to prove extravagant as well as unsatisfactory in other ways. Doubtless the committee have kept this aspect of their problem in view; and we express the earnest hope that other considerations will not be allowed to override it.

In many other ways it is clear that the management are displaying that activity without which so ambitious a proposal could never be carried through. Drawing-room meetings are being arranged, and a guild has been started to supply garments, linen, and so on, for the hospital, and to help in other ways. It may further be noted that a piece of freehold land in a suitable part of Kent, with a house on it, has been given for eventual use as a convalescent home. It will, we presume, be some considerable time before this home becomes practical politics; but it is encouraging to know that the land and house are there when the time does come. The committee will have to proceed with considerable caution, it seems to us, in the matter of finance. Encouraging as the result of their efforts so far is, the fact has to be faced that the generous anonymous donors have provided seven-eighths of the whole sum as yet obtained. If more friends can be found as benevolent and as wealthy, well and good; but the basis of financial support is not very broad at present, and the position demands circumspection in the accumulation of liabilities. It is quite true that the hospital, once in being, is itself its own best advertisement and stimulus to the subscribing public; and that a bold policy in this respect often pays far better than timidity and vacillation. But there is a golden mean between faint-heartedness on the one hand and unbusinesslike rashness on the other; if the committee can steer this discreet course, they will be entitled to every credit for a daring plan achieved without unnecessary talk and fuss in an uncommonly short space of time.

